# The effect of a neck-specific exercise program on cervical kinesthesia for patients with chronic whiplash-associated disorders: a case-control study

**DOI:** 10.1186/s12891-024-07427-9

**Published:** 2024-05-02

**Authors:** Harpa Ragnarsdottir, Gunnel Peterson, Magnus K Gislason, Gudny L Oddsdottir, Anneli Peolsson

**Affiliations:** 1 Research Centre of Movement Science, Department of Physiotherapy, University of Iceland, Stapi v/Hringbraut 31, Reykjavik, 101 IS Iceland; 2 Elja Physiotherapy, Hafnafjordur, Iceland; 3 Department of Health, Medicine and Caring Sciences, Unit of Physiotherapy, Linköping University, Linköping, Sweden; 4 Centre for Clinical Research Sörmland, Uppsala University, Eskilstuna, Sweden; 5 Institute of Biomedical and Neural Engineering, Reykjavik University, Reykjavik, Iceland; 6 Occupational and Environmental Medicine Center, Department of Health, Medicine and Caring Sciences, Clinical Medicine, Linköping University, Linköping, Sweden

**Keywords:** Movement control, Whiplash, Exercise therapy, Rehabilitation, Spine

## Abstract

**Introduction:**

Cervical kinesthesia is an important part of movement control and of great importance for daily function. Previous research on kinesthesia in whiplash-associated disorders (WAD) has focused on grades I-II. More research is needed on WAD grade III. The aim of this study was to investigate cervical kinesthesia in individuals with WAD grades II-III before and after a neck-specific exercise intervention and compare them to healthy controls.

**Methods:**

A prospective, case-control study with a treatment arm (*n* = 30) and a healthy control arm (*n* = 30) was conducted in Sweden. The WAD group received a neck-specific exercise program for 12 weeks. The primary outcome to evaluate kinesthesia was neck movement control (the Fly test). Secondary outcomes were neck disability, dizziness and neck pain intensity before and after the Fly test. Outcomes were measured at baseline and post-treatment. The control arm underwent measurements at baseline except for the dizziness questionnaire. A linear mixed model was used to evaluate difference between groups (WAD and control) and over time, with difficulty level in the Fly test and gender as factors.

**Results:**

Between-group analysis showed statistically significant differences in three out of five kinesthetic metrics (*p* = 0.002 to 0.008), but not for the WAD-group follow-up versus healthy control baseline measurements. Results showed significant improvements for the WAD-group over time for three out of five kinaesthesia metrics (*p* < 0.001 to 0.008) and for neck disability (*p* < 0.001) and pain (*p* = 0.005), but not for dizziness (*p* = 0.70).

**Conclusions:**

The exercise program shows promising results in improving kinesthesia and reducing neck pain and disability in the chronic WAD phase. Future research might benefit from focusing on adding kinesthetic exercises to the exercise protocol and evaluating its beneficial effects on dizziness or further improvement in kinesthesia.

**Impact statement:**

Kinesthesia can be improved in chronic WAD patients without the use of specific kinesthetic exercises.

**Trial registration:**

ClinicalTrials.gov (NCT03664934), first registration approved 11/09/2018.

## Introduction

Neck pain is a costly and common health problem which can be of insidious onset or can follow a trauma [1, 2]. Moreover, neck pain seems to be one of the most common complaints of patients with Whiplash Associated Disorders (WAD) [3]. Researchers have found that up to 50% of those diagnosed with WAD will recover within 3 months, the rest will have continuous symptoms resulting in chronic WAD [4].

The cervical spine, especially the upper cervical spine, is a very delicate sensory organ due to its direct neurophysiological connections to vital organs and functions in the head [5–8]. As a consequence, the cervical spine is an extremely vulnerable structure and source of a plethora of symptoms e.g., dizziness [9] and visual problems [10]. In regard to anatomy, it is apparent that the cervical spine is heavily dependent on muscular support [11–14], whereas the deep muscles are particularly important for cervical stability [12, 15, 16]. Furthermore, it has been found that muscle function and joint position are important for postural and motor control (i.e., interactions between vision and vestibular systems) [17, 18].

Kinesthesia can be defined as a sensation which detects and discriminates between the relative weight of body parts, joint positions and movements, including direction, amplitude and speed [19]. This term, therefore, includes qualities that are supposed to be a result of proprioception (proprioception defined as the awareness of joint position) [20] and is important to daily function. Kinesthesia can be tested actively in a clinical setting [21] and may be of importance for improved diagnostics and directions of rehabilitation. It is, therefore, the most appropriate term in clinical measurements for altered cervical proprioceptive function. The proprioceptive mechanisms controlling the head on the body have been tested clinically by simple target-matching tasks [22–24] and by virtual reality [21, 25–30], which may be a more functional testing of kinesthesia in several directions. Most studies include chronic neck pain patients in general, but kinesthesia has been measured in individuals with WAD as well [31, 32], although individuals with severe (WAD grade III) chronic WAD have been excluded. More research is especially needed for this vulnerable group.

Research has been done on the effect of neck-specific exercises on both subjective symptoms such as headaches and dizziness, and on objective measures such as proprioception in patients with neck pain, with promising results [25, 27, 28, 33, 34]. A recent study compared a neck-specific exercise program consisting of eye-head coordination and isometric deep neck muscle exercises to a general neck exercise program consisting of free range of motion and shoulder shrug for patients with chronic non-specific neck pain. They found that individuals in both groups showed significant improvements in proprioception [35] suggesting that exercises focusing on strength and range of motion can affect the awareness of posture. To the researchers knowledge, the effect of neck specific exercises without proprioceptive or kinesthatic training, i.e. a program consisting of facilitation of the deep cervical muscles, on an improvement in cervical kinesthesia has not yet been evaluated.

The aim was to investigate cervical kinesthesia (i.e., movement control in real-time during active movements [21]) in individuals with chronic WAD before and after a neck-specific exercise intervention that focuses on facilitation and strengthening of the deep cervical muscles [36], and to compare them to healthy controls. Subjects are expected to have worse cervical kinesthesia compared to healthy controls and to show an improvement following the intervention.

The hypothesis was that individuals with WAD may have worse outcome compared to healthy controls and that the WAD group will approve after the intervention.

## Methods

### Design

A prospective, case-control study with a treatment arm consisting of 30 individuals with chronic (≥ 6 months duration) WAD (WAD group), grade II (neck pain and clinical musculoskeletal signs) and grade III (grade II plus neurological signs), receiving neck-specific exercise treatment program according to guidelines, and a control arm consisting of 30 healthy individuals (control group) (Tables 1 and 2). Participants for the treatment arm were recruited through healthcare providers, advertisements in newspapers, posters, social media, and the university’s website. Interested individuals contacted the research team through the project website. Following completion of a short on-line survey, a project team member (physical therapist [PT]) conducted a telephone interview, asking about medical history. Arranging an appointment for a physical examination and an additional interview was set up as the last step to ensure that the criteria for study participation were met. When it was apparent that all the study criteria were met, and written and oral informed consent were confirmed, participants completed a written questionnaire and had their baseline measurements taken [36]. Data collection was in the form of subjective questionnaires and objective tests of physical neck-related function at baseline (before randomization) and after 3 months (end of physical therapy rehabilitation).

**Table 1:** Background variables and means, standard deviation, median and interquartile range for subjective measurements

Background variables			Treatment group			Healthy controls (HC)					
Gender, female, n (%)			23 (76.7%)			23 (76.7%)					
*Educational level, n (%)*											
Elementary school			0 (0)			NA					
High school			10 (33.3)			NA					
University			19 (63.3)			NA					
Other			1 (3.3)			NA					
*Marrital status*											
Living alone			6 (20)			NA					
Married or cohabitian			20 (66.7)			NA					
Other			4 (13.3)			NA					
Use of analgesic drugs for neck pain, yes (%)			23 (76.7)			NA					
Smokes, yes (%)			2 (6.7)			NA					
***Outcome measures***	**Baseline (BL)** ***Mean (SD)/median (IQR)***	**Follow-up (FU)** ***Mean (SD)/median (IQR)***			**Healthy Controls (HC)** ***Mean (SD)/median (IQR)***				***p value***		
									**BL vs. HC**	**FU vs. HC**	
**NDI**	19.6 (7)/18.5 (13-24.8)	15.1 (7.9)/16 (9–21)			0.6 (0.8)/0 (0–1)				< 0.01	< 0.01	
Pre-VAS	43.4 (22)/42.5 (25-58.8)	26.5 (23.7)/19 (4.5–46)			0.1 (0.5)/0 (0–0)				< 0.01	< 0.01	
Post-VAS	52.6 (21.6)/54 (39.3–68)	34 (26)/40 (9–53)			1 (2.7)/0 (0–0)				< 0.01	< 0.01	
DHI	33.2 (20.3)/30 (18–46)	25.9 (22.2)/19 (10.5–41.5)			NA						

* SD: standard deviation; IQR: interquartile range; BL: baseline; FU: follow-up; HC: healthy controls; NDI: Neck Disability Index; Pre-VAS: Visual Analog Scale (VAS) pre butterfly measurement; Post-VAS: VAS post butterfly measurement; DHI: Dizziness Handicap Inventory

**Table 2:** Physical pathology leading to exclusion

	Treatment group	Healthy controls
Inclusion	● Chronic (> 6 months, < 5) neck problems ● WAD grades II-III [59] verified by clinical examination ● Average neck pain ≥ 2/10 on the Visual Analogue Scale (VAS) [60] last week prior to examination ● ≥20% neck disability on the Neck Disability Index (NDI) [41] ● of working age (18– 63 years) ● within daily reach of a computer/tablet/smartphone and Internet ● shown to have exhibited neck symptoms within the first week following the injury (i.e., neck pain, neck stiffness, or cervical radiculopathy) ● righthanded in addition to experiencing either equal-sided or dominant right-sided pain	● age and gender matched ● healthy individuals ● without neck pain or disability (VAS < 10 mm, NDI < 5%) ● without known diseases
Exclusion	● exhibiting signs of head injury* at the time of WAD injury ● previous fractures or dislocation of the cervical spine ● considerable degree of known/suspected physical pathology** ● severe neck problems within their medical history which resulted in sick leave for more than a month in the year before the current whiplash injury ● generalized or more overwhelming pain occurring elsewhere in the body presently ● other illness/injury that may prevent full participation from being feasible ● lack of ability to either understand or write Swedish ● increased risk of bleeding ● severe obesity (body mass index; BMI > 35) ● contraindications of MRI [48]	● earlier neck injury ● recurrent neck pain ● earlier treatment for neck pain ● increased risk of bleeding ● BMI > 35 ● contraindications of MRI [48]

*Signs of head injury: amnesia before or after injury, loss of consciousness, altered mental status (e.g., confusion, disorientation), focal neurological changes (changes in perceptions of smell and taste)

**known or suspected physical pathology included: myelopathy, spinal tumours, spinal infection, ongoing malignancy, cervical spine surgery

**Fig. 1:**
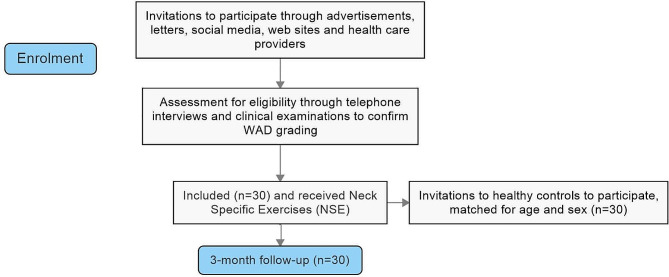
Flow-diagram of trial protocol

Healthy individuals were recruited consecutively among friends, family, University/ hospital staff and through advertisement on social media to match the age and gender of a participant in the treatment arm in order to compare cervical kinesthesia of the WAD group to healthy controls (Fig. 1). Inclusion and exclusion criteria for each group can be seen in Table 2. Independent PTs in primary care distributed the treatment for the treatment/WAD group. To ensure un-biased data collection, the project manager was not involved in the collection of data. The physical measurements were performed by independent test leaders/specially trained, skilled, PTs. The Regional Ethical Review Board in Linköping, Sweden (dnr 2016/135–31 and 2017/556–32) approved the project. The protocol was registered before data collection started (Clinicaltrial.gov Protocol ID: NCT03664934, first registration approved 11/09/2018).

The participants were recruited from 10 county councils in Sweden between October 4, 2018 to December 16, 2021. Intervention was performed in outpatient care in Sweden. All measurements were performed at Linköping University, University Hospital movement laboratory, Linköping, Sweden. There was no harms or unintended effects, except for muscle soreness after exercise.

The trial ended when all participants, 30 in each group, had been recruited and all measurements had been performed.

### Neck-specific intervention

The treatment consisted of the same neck-specific exercise program and information but distributed in two different ways. The neck-specific exercise (NSE) group (*n* = 6) exercised twice a week at a PT clinic for three months. (The first visit was a 60-minute examination, including information and the first exercise session, and the remaining sessions were 30-minute exercise sessions.) The neck specific internet-based exercise (NSEIT) group (*n* = 24) had four PT visits only, combined with a web-based system [37]. (The first PT visit was the same as for the NSE group, and the remaining three PT visits were 30-minute sessions at weeks 2, 3 and 7 for follow-up and progression/adjustment of exercises.) A computer-based block randomization list stratified by sex was used for randomization to the 2 groups. The groups were originally meant to include 15 individuals each, but due to the Covid-19 pandemic, researchers were not able to continue with recruitment for NSE treatment. The exercise program (both groups) focused on facilitation of the deep cervical muscles, starting with neuromuscular training of the deep neck muscles in supine position, performed at home 2x/day. After 2–3 weeks, exercises were progressed to a sitting position and resistance with rubber bands was used, aiming to increase neck muscle endurance. The exercise program was individualized, and some participants needed more time in the first supine phase. Information regarding exercises was given with the help of an internet support system outside the healthcare system (NSEIT group) or at the PT appointments (NSE group). Photos and videos of the exercises, information, and answers to frequently asked questions were available on the internet for the NSEIT group. (This was a web-based system designed by the project leaders at the university.) Participants in both groups were required to keep an exercise diary via the system. The system was programmed to automatically send text message reminders if participants failed to complete their exercise diary in full. Participants were able to contact the corresponding physical therapist if necessary. Explanations and justification for the exercises included basic information about the musculoskeletal anatomy of the neck that was relevant to the exercises, designed to motivate them and help make them feel safe and reassured. Elements of a behavioural approach were included, such as neurophysiological and neurobiological education and strategies for dealing with neck pain relapse. Exercises were individually adjusted according to the individual’s physical conditions and progressively increased in frequency and intensity. Exercise-related pain provocation was not accepted. The exercises had been used with good results in a previous RCT [33, 38]. Participants were asked to not seek other health care for their WAD (especially physical therapy) during the study period.

### Outcome measures

All measurements were collected by experienced test-leaders, registered health-care personal and researchers that monitored the data and guaranteed high quality data. Adverse events and harms were registered by the test-leaders. A research assistant phoned individuals that did not appear for a PT appointment. Data were part of the Health Secrets Act (Swedish law) and were stored at Linköping University, Sweden. The project leaders had access to the final trial dataset. Measurements were performed at baseline (both groups) and at 3-month follow-up when the intervention in the study ended (WAD-group only).

The following subjective and objective outcome measures were collected for the treatment group:

### Objective outcome measures, using NeckCare

**Fig. 2:**
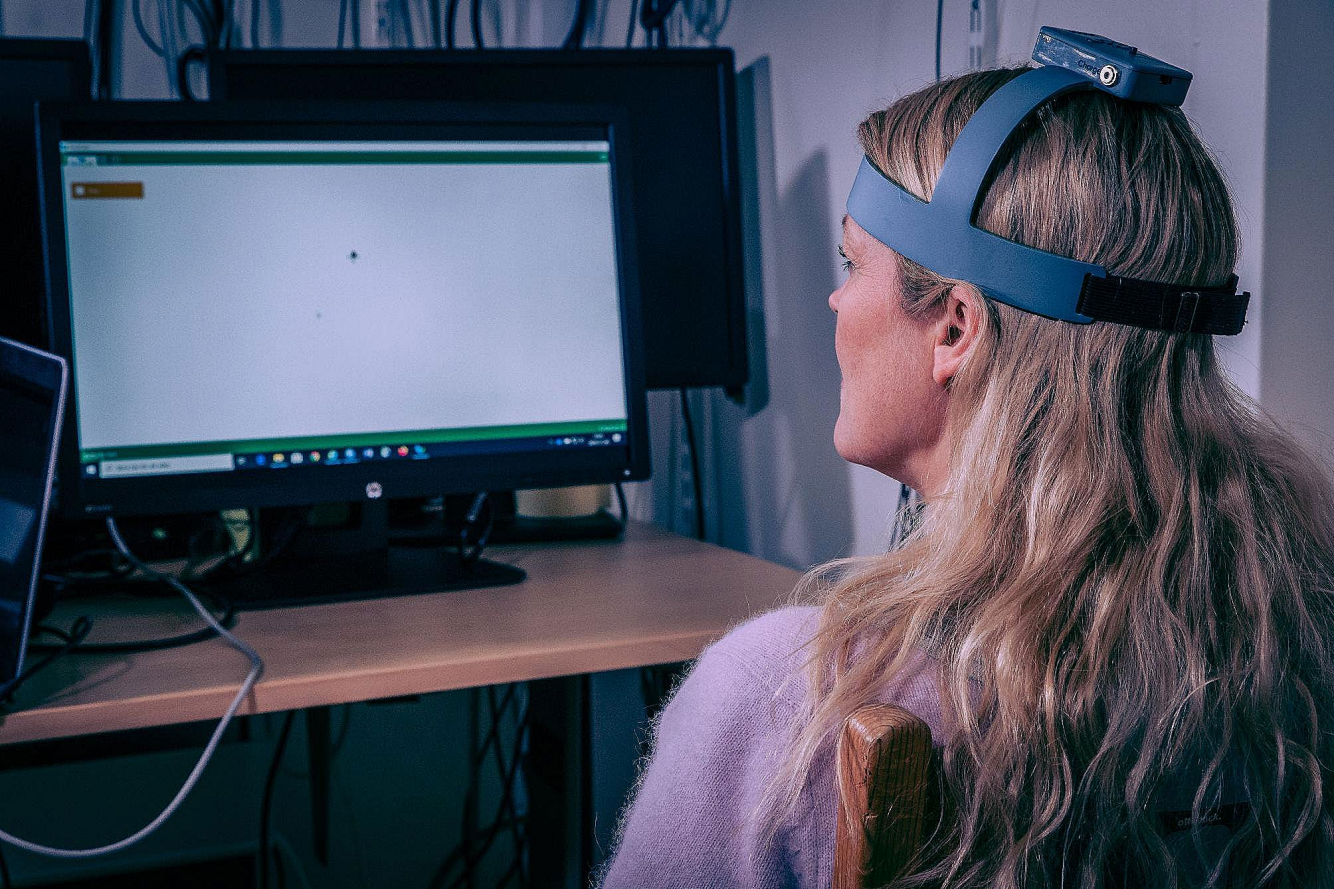
Experimental set-up. (The photo is published with consent from the woman on the photo (the woman is the last author)

For objective measures, the NeckCare system (NeckCare inc., Reykjavík, Iceland) was used. The equpment consists of a neck gear/plastic helmet, with a 3D accuracy orientation sensor that tracks the cervical position sense in space. Participants were sitting in a good postural position (slight support for the low back) with the thoracic spine fixated with a strap at T4 level, neck in neutral position, hands on thighs, thighs apart, 90 cm in front of a computer screen, with the helmet on their head as can be seen in Fig. 2. The system was used to measure neck movement control during the Fly test, which has been proven to be a reliable and a valid measure for assessing movement control of the cervical spine [21]:

- *Fly Test* [21, 39, 40] Procedure for the main outcome Fly Test: The participant tracked an unpredictable path as accurately as possible using head movement to manipulate the on-screen cursor. Three different trajectories with increasing difficulty (easy, medium and difficult) determined by the geometry of the movement tasks, the velocity of the target and the length of the trajectories, as described by Oddsdottir et al. [40], appeared on the screen. Each pattern was repeated three times in random order (a total of nine movement patterns). Before each pattern the system would count down from 3 to 1. Participants were told to move their head and neck once in a left to right rotation between patterns and then go back to their neutral neck position before the next pattern appeared on the screen. Participants were asked to do their best but not otherwise encouraged. A pre-test trial was performed to familiarize participants with the test procedures.
- Metrics: 1) Amplitude Accuracy (AA): the absolute average distance (radius) in arbitrary length units between the cursor that represents the head position and the target, where a lesser value represents a better score; 2–4) Directional Accuracy: the percentage of time the cursor that represents the head position spends in a mathematically determined, invisible free zone around the target (measured as time on target, [TOT] where a greater value indicates a better score) and the percentage of time the cursor is ahead of the free zone (measured as overshoot, [OS] where a lesser value indicates a better score) or behind the free zone (measured as undershoot, [US] where a lesser value indicates a better score); 5) Smoothness of Movement Index (SMI): The index is calculated based on the third positional derivative with respect to time and normalized against the trajectory of the target. The SMI is therefore a representation of the jerkiness of the movement and is scaled between 0 and 5 with 0 being the best and 5 being the worst.
- Metrics 1–4 show independent values for each of the three difficulty levels. Metric 3 shows value for the easy trajectory alone.

### Participant background data and outcome measures

Participant background data included personal details, neck pain intensity and functional difficulties due to dizziness and information about other diseases and medication and patient-reported neck specific disability. Data were collected through questionnaires on Linköping University´s website Survey and Reports.

1. Neck pain intensity, measured by a 100 mm Visual Analog Scale (VAS) with 0 mm representing “no pain” and 100 mm representing the “worst pain imaginable” [41, 42], was collected before (Pre-VAS) and after (Post-VAS) as objective measurements for both groups.
2. The Neck Disability Index (NDI) [43] is a 10-item self-report questionnaire with good validity and reliability [44–46], scored on a 0 to 5 rating scale, where a higher score represents more neck pain related disability.
3. Dizziness Handicap Inventory (DHI) is a 25-item self-assessment inventory to evaluate the self-perceived handicapping effects imposed by dizziness [47], scored on a 0 to 4 rating scale, with a higher score representing more self-perceived handicap.

Measurements were carried out at baseline and at a three-month follow-up (end of treatment) for the patient group. For the age- and gender-matched healthy volunteers the following measurements were collected without any follow up: 1–3 (pain on VAS, Borg, and NDI), age, gender, height, weight, physical activity, other diseases, and use of analgesics were collected.

### Data analyses and statistics

Data were analysed in the statistical software R and in Jamovi in collaboration with a statistician, independent of sponsors and competing interests. Earlier studies have shown that 30 individuals are enough for comparisons between individuals with health problems and healthy controls [11, 16, 34, 39, 48–50]. A sample size calculation was not performed as this is the first study investigating the effects of individuals with WAD grade II and III after a neck-specific exercise program using the Fly test, i.e. assumptions about calculation would have consisted of guesswork. The two treatment groups (NSE, NSEIT) were treated as one combined exercise group (WAD group). Mixed models were used to compare differences in objective measurements (four metrics on the Fly test; AA, TOT, US and OS) for the WAD group over time (baseline versus follow up) with difficulty level on the Fly test and gender as factors, and to compare between group differences with difficulty level on the Fly test and group (WAD or control) as factors. For the between group differences, one model was made for baseline WAD-group measurements versus the control group to assess differences in kinesthesia between injured and uninjured, and another model for follow-up WAD-group measurements versus control group to assess if there was still a difference between the groups following the treatment period for the WAD group. Background data were evaluated by descriptive statistics. Differences in objective measurements and in the third Fly metric (SMI) were determined using paired t-tests (mean and standard deviation) for difference over time evaluation, and t-tests for treatment versus control group evaluation. A Wilcoxon test (for between group evaluation) or paired Wilcoxon tests (for difference over-time evaluation) were used when appropriate. The statistically significant value was set at *p* = 0.05. Pearson correlation coefficients were used to determine correlations between changes seen in questionnaire data (NDI, DHI, pre-VAS and post-VAS) and kinesthesia performance (AA, TOT, OS, US, and SMI) across the study period for the WAD group.

## Results

A total of 30 individuals with chronic WAD grade II or III, mean age 43 years (SD 10.72, range 20–60) and 30 age- and gender-matched healthy controls, mean age 43 years (SD 10.8, range 20–61) were recruited between October 4, 2018 and December 16, 2021. At baseline measurements, 46.7% of individuals in the treatment group were classified with having WAD grade II (*n* = 14) and the rest (*n* = 16) into the WAD grade III. At follow-up measurements, 63.3% (*n* = 19) had WAD grade II while the rest (*n* = 11) were classified as having WAD grade III. According to the exercise diary, compliance to the exercise program was 80–100% for 60% of the individuals (*n* = 18), 50–79% for 26.7% (*n* = 8) and 20–49% for 13.3% (*n* = 4). Average number of days between baseline and follow-up measurements for the treatment group was 147.5 days (range 91–316), but should have been 90 days. Due to the Covid-19 pandemic restrictions the test leaders were, for a period, not allowed to see participants for the lab tests. Tables 1 and 3 show baseline characteristics and mean scores in subjective and objective measurements.

**Table 3:** Means, standard deviation, median and interquartile range for objective measurements

Outcome measures	Baseline Mean (SD)/median (IQR)	Follow-up Mean (SD)/median (IQR)	Healthy Controls Mean (SD)/median (IQR)
Butterfly (AAe)	2 (0.8)/1.8 (1.5–2.2)	1.7 (0.5)/1.6 (1.4–1.9)	1.5 (0.3)/1.5 (1.3–1.7)
Butterfly (AAm)	3.4 (1.3)/3.1 (2.7–3.5)	3 (0.8)/2.8 (2.5–3.6)	2.7 (0.5)/2.6 (2.3–2.9)
Butterfly (AAd)	5.3 (1.9)/4.8 (4-6.4)	4.7 (1.5)/4.2 (3.9-5)	4.1 (0.6)/4 (3.7–4.3)
Butterfly (TOTe)	66.6 (13.3)/70.1 (63.3–76.1)	72.1 (10.2)/73.1 (67.5–78.5)	73.5 (6.4)/74.3 (70.2–79)
Butterfly (TOTm)	36.4 (10.8)/37.3 (29.4–44)	41.3 (12.6)/40.6 (31.1–47.7)	44.1 (9.7)/44.4 (38.6–50.5)
Butterfly (TOTd)	18.1 (7.2)/19 (12-23.7)	20.9 (8)/20.9 (17.5–23.1)	22.5 (5.4)/23.5 (20.6–25.9)
Butterfly (OSe)	7.3 (4.2)/6.1 (5-8.2)	6.6 (3.8)/6.4 (3.5–8.9)	5.7 (2.8)/5.1 (3.6-8)
Butterfly (OSm)	16.6 (7.6)/14.8 (11.9–19.6)	14.8 (6.2)/14.6 (9.4–18.2)	14 (5.9)/12.4 (9.5–18.3)
Butterfly (OSd)	17 (8)/16.5 (10.8–19.8)	17 (7.1)/14.8 (11.1–22)	16.2 (6.7)/14.8 (11.9–20.1)
Butterfly (USe)	26 (10.7)/24.5 (18.1–30)	21.3 (8.1)/21.9 (15.6–24)	20.8 (6.3)/18.8 (17-23.5)
Butterfly (USm)	47 (9.6)/46.4 (41.9–51.3)	44 (10.8)/44.1 (36.8–52)	41.9 (9.4)/39.8 (36.5–47.6)
Butterfly (USd)	64.8 (7.4)/63 (60-68.2)	62.1 (7.4)/63 (58-66.5)	61.4 (6.8)/61.2 (58.3–64.6)
Butterfly (SMI)	2.0 (0.7)/1.8 (1.50–2.33)	1.9 (0.59)/2.0 (1.52–2.02)	2.2 (0.53)/2.3 (1.90–2.54)

*SD: standard deviation; IQR: interquartile range; AA: Amplitude Accuracy; e: easy; m: medium; d: difficult; TOT: Time on Target, OS: Overshoot, US: Undershoot, SMI: Smoothness of Movement Index

### Between-group differences (WAD versus healthy controls) in the fly test

There were significant differences between groups (WAD and controls) at baseline measurements for AA (F(1,73.7) = 10.61, *p* = 0.002), TOT (F(1,75.1) = 9.52, *p* = 0.003) and US (F(1,68.9) = 7.45, *p* = 0.008) with the control group showing a better score. A significant difference was not found for OS (F(71.3) = 1.69, *p* = 0.197). In addition, a t-test showed no between-group differences on SMI (*p* = 0.08). A significant difference was found between difficulty levels for all metrics (*p* < 0.001), except for SMI which was not measured independently for each difficulty pattern. A significant difference was found for difficulty level and group interaction for AA (*p* = 0.001) and the post-hoc test showed that the significant difference was only for the difficult path (*p* = 0.001).

No significant between-group differences were found between the WAD groups’ 3 months measurements and the baseline measurements for the healthy controls. For subjective variables, significant differences were found beween all baseline and healthy controls and between follow ups and healthy controls (*p* < 0.01) (Table 1).

### Differences over time (baseline versus follow up measurements) for the WAD-group

**Fig. 3:**
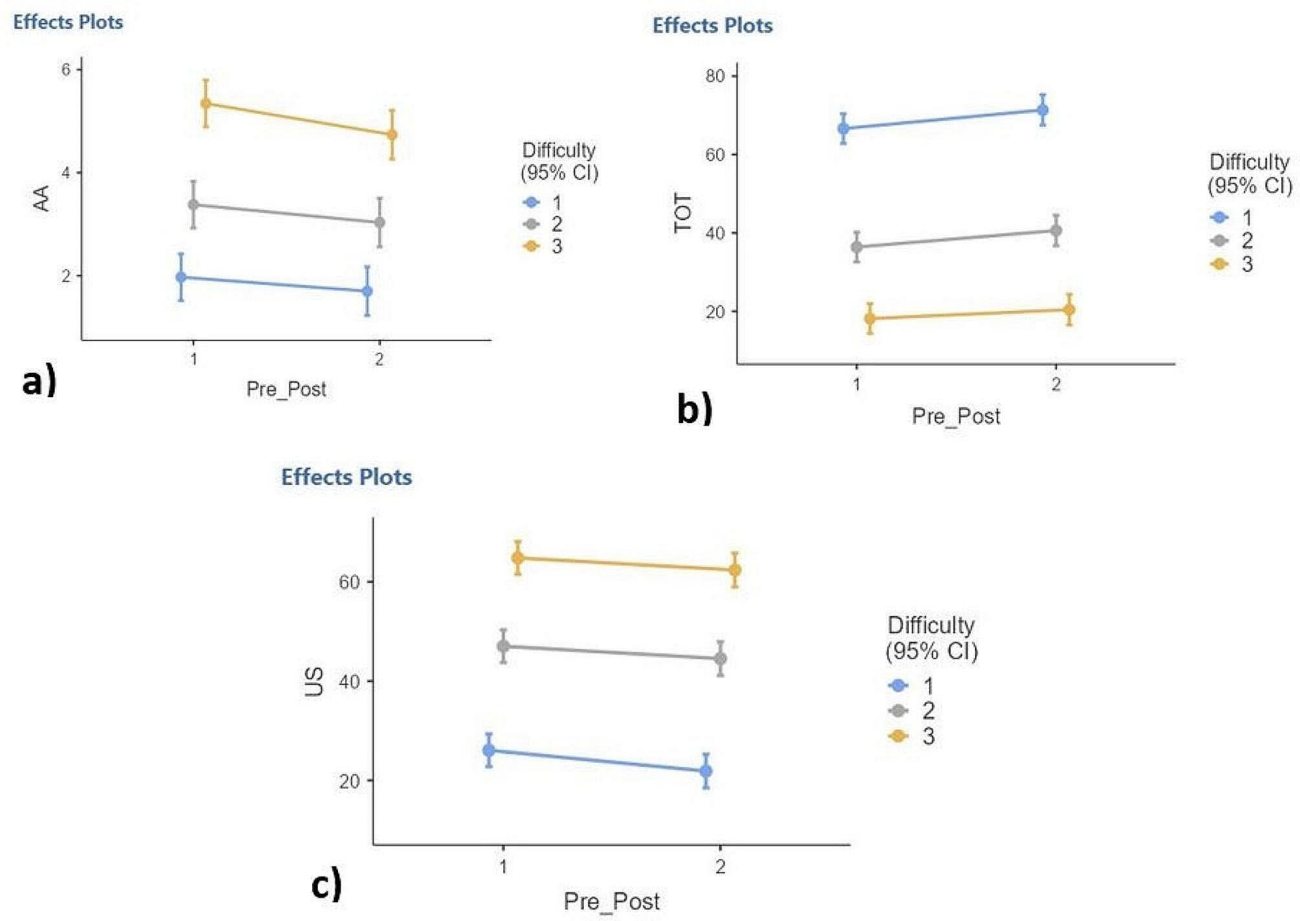
The Fly test: Response over time by difficulty level. The figure shows the response over time by difficulty level for three metrics: **(a)** Amplitude Accuracy, **(b)** Time on Target, and **(c)** Undershoot. The X-axis shows mean values for each difficulty level pre (1) and post (2) treatment period

A mixed model evaluating differences in means in objective measurements over time with difficulty level as a factor showed statistically significant improvement over time for AA (F(1,112) = 7.24, *p* = 0.008), TOT (F(1,111.4) = 14.41, *p* < 0.001*)* and US (F(1,111.2) = 11.25, *p* = 0.001) (Fig. 3), but not for OS (F(1,110) = 1.14, *p* = 0.287). A t-test showed no difference over time for SMI (*p* = 0.53). A statistically significant difference was found between difficulties for all Fly metrics (*p* < 0.001) except for SMI which was not measured independently for each difficulty pattern. No statistical differences were seen with difficulties for the Fly metrics and time interaction (*p* = 0.661 to 0.808).

For subjective measurements, a significant improvement was found over time (Table 1) (*p* < 0.001) and a significant difference was found between genders with males showing better scores for NDI (*p* = 0.020), but no statistical difference was seen with gender and time interaction. Finally, a statistically significant difference was found over time for Pre-VAS (*p* = 0.005) and Post-VAS (*p* < 0.001), but not between genders or with gender and time interaction. No statistical difference was found over time or between genders for the DHI (*p* = 0.70).

### Correlation

When correlations between kinesthetic metrics and neck disability, dizziness and pain were assessed for baseline measurements, significant moderate correlations were found between the AA difficult path and the Pre-VAS score (*r* = 0.44, *p* = 0.02) and the Post-VAS score (*r* = 0.44, *p* = 0.02), and between the US difficult path and Pre-VAS score (*r* = 0.41, *p* = 0.04) and the Post-VAS score (*r* = 0.44, *p* = 0.02). When the correlations between kinesthetic metrics and neck disability, dizziness and pain were assessed for follow-up measurements, significant weak-moderate correlations were found between the US medium path and the Post-VAS score (*r* = 0.39, *p* = 0.04) and between the US difficult path and Post-VAS score (*r* = 0.45, *p* = 0.01).

## Discussion

Results of this study show a significant between group (WAD versus controls) difference at baseline measurements for three (AA, TOT, US) out of five kinesthetic metrics, with the WAD group showing worse scores than the control group (Table 3). No between group differences were found for follow up kinesthetic metrics measurements. Difference over time analysis (WAD: baseline versus follow up) showed a significant improvement over time for three (AA, TOT, US) out of five kinesthetic metrics. Researchers have found some correlation between poor kinesthesia and neck pain [22] and it has been suggested that cervical kinematics should be evaluated clinically in patients with neck pain to direct treatment strategies [23]. The results of this study suggest that the neck-specific exercise program had some beneficial effect on kinesthesia for chronic WAD patients despite the lack of kinesthetic exercises in the program. This might be due to possible improvement of the function of deep neck muscles, which participants work on in week one of the exercise program. Through improvements in technology [51, 52], it has already been seen that the deep neck muscles, which contain a lot of proprioceptors [53] that are important for kinesthetic funcion [54], are impaired in WAD [55] and, furthermore, they can be improved via neck-specific exercise [34]. In addition, deep cervical fascilitation has been shown to improve joint position sense [56].

For subjective measurements, differences between baseline and follow up measurements were statistically significant for reduction in neck pain and disability, as has been shown in previous research [38]. However, no statistically significant difference was found for the Dizziness Handicap Inventory, suggesting sensorimotor function and neck muscle endurance exercises alone do not affect dizziness in patients with chronic WAD. This is, however, in contrast to previous research that have shown improvements using neck-specific exercise on dizziness for patients with chronic WAD [57], as well as patients with other conditions related to the neck such as cervical radiculopathy [58]. The differences may be due to different study criteria, measurements used, and sample size.

Interestingly, correlational analysis showed correlations between two kinesthetic metrics (AA difficult and US difficult) to both Pre- and Post-VAS in baseline measurements, with a higher correlation between Post-VAS and US difficult, while two metrics (US medium and difficult) in the follow-up measurements were found to correlate with Post-VAS score. This might suggest that the Fly test is triggering or increasing pain levels with participants. A possible resason could be that individuals who are lacking movement control might be overactivating bigger muscle groups of the neck, thus increasing or triggering a pain response.

Other research has shown meaningful change in kinesthesia following a kinesthesia exercise program [24, 30]. The exercise program in this study shows promise in improving kinesthesia and reducing neck pain and disability in the chronic WAD phase. However, future research might benefit from focusing on adding kinesthetic exercises to the exercise protocol and evaluating its beneficial effects on dizziness or further improvements in kinesthesia. Local neck treatment might also be beneficial to this program as local neck treatment has been shown to reduce dizziness in individuals with neck pain [59, 60].

### Trial limitations

Due to the Covid-19 epidemic, researchers were not able to recruit 15 into the NSE group and 15 to the NSEIT group as was the original protocol, resulting in 80% of the treatment group being treated primarily remotely. However, NSEIT was non-inferior to NSE and demonstrated sustained clinically important changes in disability and pain for approximately 50% of patients with WAD grades II and III [61]. Authors believe that neck-specific exercises also will improve kinesiophibia to the same extent regardless of the different way to deliver the exercises. Both WAD-groups received the same exercises and the same information, although distributed differently. There were no significant differences in outcome between the two groups (NSEIT vs. NSE) in other variables such as pain and disability [61]. For interpretation of results, researchers had to trust participants to keep an honest exercise diary. A total of 77% of participants in the injured group were female, leaving only seven males in this group. The low proportion of males might have skewed results in regard to male-female differences in means of measurements, but gender proportion was in line with earlier studies regarding WAD [33, 38, 57]. Finally, the control arm only underwent measurements once, making impossible an analysis over time to evaluate the learning effect of the Fly test for the control group. Learning effect from clinical testing have been well documented and has for example been shown to maintain during a 2-month period in the 6-minute walking test [62]. Therefore, it would have been of great value to test the control group again in order to account for the learning effect of the Fly test.

## Conclusions

The exercise program shows promising results in improving kinesthesia and reducing neck pain and disability in the chronic WAD phase. Kinesthesia can thereby be improved in chronic WAD patients without the use of specific kinesthetic exercises. Future research might benefit from focusing on adding kinesthetic exercises to the exercise protocol and evaluating its beneficial effects on dizziness or further improvement in kinesthesia. The results may be generalizable to participants with WAD II and III.

## Data Availability

Upon reasonable request to Anneli Peolsson and after ethical permission.

## Abbreviations

WAD: Whiplash Associated Disorders
PT: Physical Therapist
NSE: Neck-Specific Exercise
NSEIT: Neck-Specific Internet-based Exercise
AA: Amplitude Accuracy
TOT: Time on Target
OS: Overshoot
US: Undershoot
SMI: Smoothness of Movement Index
VAS: Visual Analog Scale
NDI: Neck Disability Questionnaire
DHI: Dizziness Handicap Questionnaire
